# Retraction: LacI-Family Transcriptional Regulator DagR Acts as a Repressor of the Agarolytic Pathway Genes in *Streptomyces coelicolor* A3(2)

**DOI:** 10.3389/fmicb.2022.942458

**Published:** 2022-05-26

**Authors:** Frontiers Editorial Office

Following publication, the corresponding author contacted the publisher after discovering the datasets of Figure 5 were fabricated. An investigation was conducted that determined the figure was indeed fabricated, as such the article is being retracted.

The authors concur with the retraction and sincerely regret any inconvenience this may have caused to the reviewers, editors, and readers of Frontiers in Microbiology.

